# The BrainWaves study of adolescent wellbeing and mental health: Methods development and pilot data

**DOI:** 10.1371/journal.pone.0338009

**Published:** 2025-12-02

**Authors:** Ryan D. Parsons, Sarah Bauermeister, Julian Turner, Natalie Coles, Simon Thompson, Emma Squires, Tracey Riseborough, Joshua Bauermeister, Abbie Simpkin, Naomi French, Shankly Cragg, Hazel Lockhart-Jones, Olly Robertson, Abhaya Adlakha, Ian Thompson, John Gallacher

**Affiliations:** 1 Department of Psychiatry, University of Oxford, Oxford, United Kingdom,; 2 The Day News & Media Ltd., London, United Kingdom; 3 Department of Data Science, Swansea University, Swansea, United Kingdom; 4 Department of Education, University of Oxford, Oxford, United Kingdom; University of Pavia: Universita degli Studi di Pavia, ITALY

## Abstract

Adolescent mental health and wellbeing are of growing concern globally with increased incidence of mental health disorders in young people. BrainWaves provides a framework for relevant and diverse research programmes into adolescent mental health and wellbeing that can translate into practice and policy. The research programme is a partnership with schools centred on establishing a large (n > 50,000) cohort and trials platform. Reported here is the BrainWaves cohort pilot study. This was designed as proof-of-concept for our recruitment and data capture pipelines, and for cost-modelling. A network of research schools was recruited and a computer-driven questionnaire administered. The eligible population was 16 + year olds who were attending the research schools. Of 41 research schools, 36 (88%) participated over one three-week and one four-week data collection period. From an eligible population of 33,531 young people, 16,010 (48%) attended the study lesson and created an account. Of the 16,010 (100%) who created an account, 15,444 (96%) consented to participate, 9,321 (60%) consented to linkage of research data with educational records, and 6,069 (39%) consented to linkage of research with school/college attendance data. Participants were aged 16–19 years, 59% female, and 76% White. Higher levels of anxiety and depression were found in females than males. Higher levels of media-based social networking were found in females, whereas higher levels of media-based gaming were found in males. Females were more likely to report insufficient sleep whilst males were more likely to report high levels of exercise. This study confirmed an ability to recruit at pace and scale. Whilst the response-rate does not indicate a representative sample, the demographics describe an inclusive and diverse sample. Data collected confirmed findings from previous studies indicating that the electronic data collection methods did not materially bias the findings. Initial cost-modelling suggests these data were collected for around £20 per participant.

## Introduction

Adolescence is a pivotal developmental period, marked by psychological, physical, and social changes [1], including the development of key domains such as personal identity [2] and autonomy [3]. The transition towards greater self-responsibility has lifelong health, wealth, and quality of life implications. Mental health and wellbeing during adolescence is of growing concern globally. Notably, the global prevalence of depression and anxiety disorders among young people has been estimated at up to 8% [4] and 6.5% [5], respectively, with the peak age of onset of mental health conditions in young people at 14.5 years old [6]. In the United Kingdom, reported mental health disorders have increased from 1 in 10 – 1 in 4 young people between 2017 and 2022, respectively [7]. Similar alarming increases are reported across industrialised societies and may be anticipated elsewhere. Although specific challenges will vary across regions and cultures, the need for a relevant evidence-base will not.

BrainWaves (https://brainwaveshub.org) is a response to the growing adolescent mental health challenge. Our mission is to provide data and develop evidence-based support materials that are classroom ready and designed to help young people manage their mental health and wellbeing. By bringing together scientists, informaticians, educators, and young people themselves, BrainWaves provides a framework for a relevant and diverse research programme that can translate into practice and policy.

The BrainWaves programme is centred around a large cohort study (n > 50,000), which aims to more closely identify the determinants, mediators and outcomes for mental health and wellbeing. Adolescence is a complex journey and a more complete understanding requires population-level longitudinal data that maps the trajectory of mental health and wellbeing, and their causes, over time. Population-level data is immediately relevant to all young people, not just those requiring specialist support. A population-wide improvement in mental health and wellbeing would also reduce the number of young people requiring specialist support. It is anticipated that data collected via the cohort study platform will provide the wider scientific community with opportunity to develop their own research agendas. Researchers around the world will have the ability to access this rich dataset in a secure informatics environment; the BrainWaves Informatics Hub (https://brainwaveshub.org/for research).

Nested within the cohort study is a trials platform. Limitations in the cohort design include the observational nature of the data, which limits causal inference. The trials platform allows hypotheses generated by observation to be tested by intervention. BrainWaves participants are consented for recontact for trials and other sub-studies, with cohort data used for participant stratification per hypothesis. Adapting the classic cohort design in this way increases rigour (targeted recruitment), reduces time (rapid recruitment), and reduces cost (reduced screen failure).

A goal of the BrainWaves cohort design is recruitment and assessment at pace and scale. Pace reduces research-cycle times and incentivises developing cost-effective methodologies. Scale maximises scientific opportunity in terms of increased heterogeneity and statistical power, and provides a large and diverse recruitment pool for the trials’ platform. These are major methodological challenges. In this paper we describe the BrainWaves cohort pilot study. This was designed as proof of concept for our recruitment and data capture pipelines. The present analysis investigates the performance of the recruitment and assessment pipelines.

## Methods

### Ethics

Ethical approval for this study was granted by the Central University Research Ethics Committee (Ref: R91383/RE001, amendment: R91383/RE004).

### Youth and schools’ consultation

The study was co-designed with input from a voluntary Young Person’s Advisory Group (YPAG). The YPAG was recruited primarily via Instagram or Twitter (X) advertisements, school recruitment, or word of mouth, representing a wide range of backgrounds and schools. The YPAG comprised 17 members aged 16–18 from across Great Britain. YPAG members self-identified as being male, female, or non-binary. A wide variety of ethnicities were represented including Arab, Asian, Black, Caribbean, Mixed, and White. The YPAG convened online via Microsoft Teams for 12 sessions between August 2023 and December 2024, with a facilitator and members of the research team present at each session. The YPAG provided diverse feedback and perspectives on aspects of the BrainWaves project, including refining research questions and providing feedback on the design and content of materials. YPAG members received honoraria for their participation.

Two sessions focussed specifically on the cohort study. The first session focused on the most effective approach for repeated measurement of participants over a 10-year period, as well as refining the questionnaire topics to enhance acceptability and alignment with youth priorities. Feedback from this session included: 1) confirmation of the use of email as a preferred contact/re-contact method, with mobile phone number as a backup, 2) support for asking participants to report weight and height (if known), but suggested providing reasoning for asking and including a trigger warning and safeguarding for this question, 3) provided suggestions for appropriate questions to ask to assess socioeconomic status, 4) provided information regarding what discrimination means to YPAG members and suggested a single open-response question to assess discrimination, 5) highlighted the importance of asking about sexual and romantic relationships, but suggested keeping these questions simple and short, 6) suggested asking about most important domains earlier in the questionnaire. A separate Youth Advisor contributed to planning the content of the first session and provided support during its delivery.

The second session focused on trialling the full participant journey when taking part in the survey, with YPAG members providing feedback to improve the acceptability of questionnaire items and the functionality of the platform. More specifically, feedback from this session included: 1) improvements to wording on consent form items to clarify optional statements, 2) improvements to the way the questionnaire is displayed on mobile phones, 3) improvements to functionality and appearance of the overall questionnaire, 4) minor changes to wording of questionnaire items to improve clarity, 5) praise for the inclusion of ‘contact for help’ safeguarding boxes below sensitive topics. The protocol was revised as a result of YPAG involvement and suggestions, and feedback was provided to the YPAG on how their input had shaped the study.

Schools were consulted via a teachers’ workshop. At the workshop, key details of the study were described. Teachers were then asked to comment on various aspects, including the sharing of personal data, questionnaire content, accessibility, sensitive topics, and safeguarding. YPAG and teacher feedback was used to improve the study. More specifically, teacher feedback included: 1) students are unlikely to have concerns about being asked for their contact details, 2) it would not be a problem if some students did not want to participate as other activities could be provided, 3) robust safeguarding is important (resulting in detailed safeguarding procedures developed for this study), 4) clarify that teachers can’t see participants’ responses to the questionnaire – important to clearly explain safety/pseudonymity of data in the participant information sheet, 5) students may have concerns about others seeing their answers – suggested completing on a mobile device instead of more visible school PC screen, 6) provided suggestions for small improvements to the clarity of wording for some questions, 7) carefully consider the length of the questionnaire to avoid students ‘switching off’. Teacher feedback from this session was combined with feedback from the YPAG to shape the final design of the study.

### Recruitment

The population sample comprised students aged 16 + years attending schools drawn from the BrainWaves Research Schools Network. This network consists of secondary schools and sixth form colleges across England, Wales and Scotland. The network was established through ‘The Day’ (https://theday.co.uk), a topical website for schools promoting oracy and critical thinking skills for young people. Schools were recruited to the network on the basis of participating in, and shaping, the research programme, receiving school-specific non-identifiable feedback on the data collected, and having access to BrainWaves developed educational materials. These materials included 17 full lessons for key stage 3–5, teacher notes, guidance for teaching BrainWaves, further reading, professional development webinars, and a monthly newsletter. Schools were offered access to a report following the study which would describe the outline findings and, where possible, the profile of their own students relative to the wider cohort. Each research school had a dedicated liaison manager to support them in participating in the cohort study, and in studies evaluating the quality and effectiveness of the BrainWaves lessons. Schools were asked to appoint a BrainWaves co-ordinator as a point of contact.

### Technology

All data were collected using the BrainWaves Informatics Hub. The Hub lies within the Trusted Research Environment of Dementias Platform UK’s Data Portal (https://portal.dementiasplatform.uk), which is ISO 27001 and DEA accredited; utilising the secure infrastructure and systems of UKSeRP developed by Swansea University (https://serp.ac.uk/serp-uk-trusted-research-environment). Capabilities include data capture, data processing pipelines for research readiness, and providing access for the wider scientific community.

### Procedure

Briefly, once a school had agreed to participate in the pilot study, all teachers responsible for supervising administration of the electronic questionnaire were encouraged to attend an online pre-questionnaire training session. Teachers who were unable to attend training sessions were provided with training resources (including a video) and were encouraged to watch the video and familiarise themselves with these resources prior to supervising the questionnaire.

Schools were provided with an information pack, which included an overview of the study for schools and a letter to be shared with parents to inform them of the study. Prior to participating in the questionnaire (no more than one week before administration), students took part in a teacher-led session (c15-20 minutes) where they learned about the wider BrainWaves study, what a longitudinal cohort study is, the importance of their involvement in the study, and how their data would be used and stored. This included information regarding how their confidentiality would be maintained throughout the study. Students were also provided with a copy of the participant information sheet via their school at least two working days before data collection, either by email or as a hard paper copy.

Data were collected in class as part of the Personal, Social, Health and Economic (PSHE) curriculum, or in a class gathered specifically for the purpose of data collection. At the beginning of the class, teachers were asked to read a script describing the purpose of the study, the type of questions that would be asked, and their right not to take part. Students were also provided with a second copy of the participation information sheet to review before beginning the study. To participate, students created a password protected login, provided online (written) informed consent, and then accessed the online questionnaire. Students were given 40 minutes to complete the questionnaire. Data were collected in two tranches. The first was a three-week period from 15^th^ April to 3^rd^ May 2024. The second was a four-week period from 11^th^ November to 6^th^ December 2024.

### Assessment

The questionnaire was administered electronically. The questionnaire was structured into sections covering demographics, mental health, wellbeing, lifestyle, cognition, physical health, adversity, and relationships. Participants could skip questions if they preferred not to answer.

The questionnaire had 30 modules organised into 10 themes representing 268 questionnaire items. For this report, the focus is on mental health, wellbeing, lifestyle and the use of social media. Anxiety and depressive symptoms were assessed using the Revised Children’s Anxiety and Depression Scale (RCADS) [8]. Overall wellbeing was assessed using the Short Warwick-Edinburgh Mental Wellbeing Scale (SWEMWBS) [9]. Flourishing was assessed by The Flourish Measure [10], whilst agency was assessed using the voice sub-scale of the Youth Empowerment Scale [11]. Resilience was measured using the Brief Resilience Scale [12]. For lifestyle, items were taken from the OxWell [13] and other studies assessing frequency of vaping, smoking, alcohol consumption, and exercise. Sufficient sleep was also assessed. Use of social media was reported as duration in hours/day of gaming and networking.

### Safeguarding

Due to the sensitive nature of questions relating to anxiety/depression, diet/body shape, self-harm, adversity/maltreatment, and bullying, the following safeguarding measures were taken:

1) Participants were provided with a brief description of potentially sensitive sections of the questionnaire, and reminded they could stop the questionnaire or skip questions at any time.
2) The questionnaire debrief form included signposting links to sources of support if young people felt distressed and reminded participants that they should speak to a trusted adult if they were upset by any of the questions. Additionally, contact details for the research team were provided. This information was also emailed to participants following completion of the questionnaire.
3) The questionnaire was completed within the school environment to ensure participants were not alone and that an adult was present in case they required assistance or support. To restrict questionnaire completion to within the school environment, the online portal was only accessible to participants between 7:30 and 17:30.
4) Following items on potentially sensitive topics, participants were provided with the option to flag that they would like to be contacted by a member of the study team (Chartered Psychologist). The research team responded to the safeguarding flag within five days, with up to three contact attempts being made.

If a safeguarding flag was raised, an initial email was sent to each participant. Email details were shared with the Designated Safeguarding Lead via a secure portal hosted on UKSeRP servers at Swansea University. This email offered a choice of options: 1) a letter that they could take to their GP to request further assistance from a mental health professional, 2) a phone call from an experienced member of the research team (Chartered Psychologist), 3) self-help website materials appropriate to each domain flagged, and/or 4) referral to their school safeguarding officer/lead. Participants were also asked to indicate if they had selected the flag tick box in error.

### Data processing and analysis plan

The focus of the present analysis is to investigate the performance of the recruitment and assessment pipelines. Recruitment pipeline measures are described using cell frequency. Distributions of demographics, psychological status and lifestyle are stratified by sex. Comparisons were made using two-tailed t-tests for independent groups and Chi^2^ as appropriate. Preliminary evidence on external validity was sought using bivariate linear regression. Specific scientific questions will be addressed in subsequent papers. All data were analysed using Stata SE Version 18 (StataCorp. 2019. Stata: Release 18. Statistical Software. College Station, TX: StataCorp LLC).

## Results

### Recruitment pipeline

Of 41 schools in the research network at the time of the study, 36 (88%) agreed to participate (Table 1). The schools included 14 state Sixth form colleges, 12 state secondary schools, nine independent schools, and one Local Authority maintained school. Schools were distributed across 31 different Local Education Authority areas in England and Scotland, representing diverse regions and localities.

**Table 1:** Participating schools according to legal status.

School	Type	Location	Eligible participants	Recruited (%)
1	State academy	London, England	421	243 (58)
2	State academy	Cheshire, England	449	285 (63)
3	Sixth form college	Sussex, England	3,387	2,009 (59)
4	Independent	Kent, England	291	84 (29)
5	State academy	Cheshire, England	27	4 (15)
6	Sixth form college	Manchester, England	450	99 (22)
7	Sixth form college	Sussex, England	4,692	1,623 (35)
8	Sixth form college	Bury St Edmunds, England	1,376	467 (34)
9	Sixth form college	Bury St Edmunds, England	2,169	630 (29)
10	Sixth form college	Great Yarmouth, England	2,112	1,079 (51)
11	Sixth form college	Surrey, England	3,518	1,637 (47)
12	Sixth form college	Huddersfield, England	3,932	2,743 (70)
13	State academy	Essex, England	568	330 (58)
14	State academy	Lincolnshire, England	186	76 (41)
15	State academy	Oxfordshire, England	365	243 (67)
16	Independent	East Yorkshire, England	93	44 (47)
17	State academy	Newbury, England	207	159 (77)
18	Sixth form college	Woking, England	772	513 (66)
19	State academy	Oxford, England	349	250 (72)
20	State academy	Wiltshire, England	79	67 (85)
21	Sixth form college	Eastleigh, England	962	760 (79)
22	Independent	London, England	94	83 (88)
23	State academy	Exmouth, England	147	69 (47)
24	Independent	Edinburgh, Scotland	138	21 (15)
25	Sixth form college	Hartlepool, England	701	68 (10)
26	Sixth form college	Stourbridge, England	2,462	765 (31)
27	Independent	Hampton, England	224	140 (63)
28	Independent	Sherborne, England	93	70 (75)
29	Sixth form college	Northumberland, England	592	84 (14)
30	Independent	Nottingham, England	73	59 (81)
31	State academy	London, England	54	38 (70)
32	State academy	Coventry, England	313	67 (21)
33	Independent	West London, England	120	106 (88)
34	LA Maintained	West Sussex, England	47	31 (66)
35	Sixth form college	Sunderland, England	2,018	462 (23)
36	Independent	Milton Keynes, England	50	36 (72)
**Total**			**33,531**	**15,444 (46)**

### Sample

From an eligible population of 33,531 young people, a total of 16,010 (48%) attended the assessment lesson and created an account. Of these, 15,444 consented to participate, representing 46% of the eligible population and 96% of those in attendance (Table 1). Of those who consented to participate, 9,321 (60%) consented to linkage of their research data to their educational records, and 6,069 (39%) consented to linkage of their research data to their school/college attendance data. Response was highly variable across schools. The average recruitment rate was 41% for sixth form colleges, 56% for state academies, and 62% for independent schools. However, technical issues in six schools/colleges and resource limitations in three colleges resulted in lower response rates (<30%). This variability reflected differences in school culture and study engagement. For the remainder of this paper, the sample will be considered as the 15,444 (100%) who consented to participate. For reporting simplicity, 52 participants who indicated ‘androgynous’ as their biological sex at birth are omitted from analyses of sex differences.

### Data completeness

Completion rates varied across modules. There was a trend of incremental attrition as the questionnaire progressed (Table 2), with no single module being associated with a substantial drop in response. This monotonic missingness pattern was investigated further. Using module sequence as a surrogate time variable, Cox regression of module completion rates according to sex found that males were more likely to drop out as the questionnaire progressed (HR = 1.13, p < 0.001, 95%CI = 1.192:1.321), indicating that missingness is not random.

**Table 2:** Data completeness according to module order.

Theme	Module order	Exemplar variables	Completion rates
Consent	1	Consent for participation	15,444 (100%)
Demographics	2	Age, sex, ethnicity, religion, SES	15,043 (97%)
Wellbeing	3	Quality of life	14,651 (95%)
	4	Mental wellbeing	14,219 (92%)
	5	Agency	14,110 (91%)
	6	Positive thought	14,003 (91%)
	7	Belonging	13,912 (90%)
	8	Self-esteem	13,742 (89%)
	9	Flourishing	13,595 (88%)
	10	Resilience	13,352 (86%)
Mental health	11	Depression, anxiety	12,818 (83%)
	12	Recent life events	12,861(83%)
Lifestyle	13	Sleep	12,588 (82%)
	14	Substance use	12,622 (82%)
	15	Hobbies & entertainment	12,450 (81%)
	16	Diet, body shape	12,286 (80%)
	17	Self-harm	11,840 (77%)
Cognition	18	Reasoning, emotional regulation	11,449 (74%)
Self-report health	19	Health, chronic disease, neurodiversity	11,280 (73%)
Adversity	20	Maltreatment	11,319 (73%)
	21	Discrimination	10,864 (70%)
Social relationships	22	Social media use	11,053 (72%)
	23	Sexual and romantic relationships	10,686 (69%)
	24	Connectedness	10,372 (67%)
	25	Home life	10,335 (67%)
	26	Friendships, bullying	10,057 (65%)
Miscellaneous	27	Social phobia	9,888 (64%)
	28	Fear of missing out	9,653 (63%)
	29	Concerns about research	9,841 (64%)
Debrief	30	User experience	8,572 (56%)

### Safeguarding

A total of 737 safeguarding flags were raised covering anxiety/depression (n = 322), diet/body shape (n = 183), self-harm (n = 121), maltreatment (n = 64), and bullying (n = 47). These flags were raised by 446 individual participants (3%), of whom 221 were female and 216 were male (n = 9 androgenous/sex not reported). Of these, 32 individuals (7%) requested further self-help website materials, 22 (5%) requested a letter to take to their GP, 7 (2%) requested to be referred to their school safeguarding officer/lead, and 3 (0.7%) requested a phone call. A further 76 (17%) indicated that they selected the flag in error, whilst 306 (69%) did not respond.

### Socio-demographics

Participants were aged 16–19 years (m = 17, SD = 0.74) being 59% female and 41% male. Neurodiversity was reported at 12% for female participants and 10% for male participants (p < 0.005), whilst disability was reported at 11% for both sexes (Table 3). A wide variety of faiths was represented, including atheist (32%), Christian (23%), agnostic (19%), no religion (13%), Muslim (9%). Buddhist, Hindu, Judaism, and Sikh, each represented 1% or less of participants. Describing ethnicity was more challenging due to the wide variety of groupings. Nevertheless, the sample was 76% White, 9% Mixed, 7% South Asian, 3% African/African-Caribbean, 3% other Asian, 0.5% Arab and 0.9% other. Bullying at least twice a month or more was low at around 7% for both males and females.

**Table 3:** Sample characteristics stratified by sex.

Theme	Variable	N	Males	Females	Sig
Demo-graphics	Sex (n,%)	14,780	6,033 (40.8%)	8,747 (59.2%)	<0.001
	Age (mean, SD)	14,728	16.8 (0.75)	16.7 (0.71)	<0.001
	Neurodiversity (n, %)	10,803	419 (10.1%)	809(12.2%)	<0.001
	Disability (n, %)	14,598	630 (10.6%)	946 (10.9%)	n.s
	Bullying (yes)	9,736	254 (6.9%)	462 (7.6%)	n.s
Mental health	Anxiety (mean, SD)	12,364	5.1 (3.8)	8.3 (4.2)	<0.001
	Depression (mean, SD)	12,392	5.2 (3.3)	6.8 (3.2)	<0.001
Wellbeing	Mental wellbeing (mean, SD)	13,730	21.9(3.7)	20.4 (3.1)	<0.001
	Agency (mean, SD)	10,363	10.3 (2.6)	9.9 (2.5)	<0.001
	Flourishing (mean, SD)	12,793	79.5 (18.5)	74.4 (17.6)	<0.001
	Resilience (mean, SD)	12,896	3.3 (0.7)	2.8 (0.7)	<0.001
Behaviour	Vaping (current)	12,351	911 (18.8%)	1,940 (25.9%)	<0.001
	Smoking (current)	12,375	554 (11.4%)	886 (11.8%)	n.s
	Alcohol (current)	12,374	2,670 (54.9%)	4,604 (61.3%)	<0.001
	Exercise (weekly or more)	12,191	3,637 (76.0%)	4,142 (55.9%)	<0.001
	Sleep (sufficient)	12,397	2,878 (58.9%)	3,229 (43.0%)	<0.001
	Drugs (once or more)	12,221	951(19.8%)	1,220 (16.5%)	<0.001
Social media (>4 hrs/day)	Time networking	10,604	1,148 (28.4%)	2,259 (34.4%)	<0.001
	Time gaming	9,959	668 (17.3%)	264 (4.3%)	<0.001

### Mental health and wellbeing

**Fig 1:**
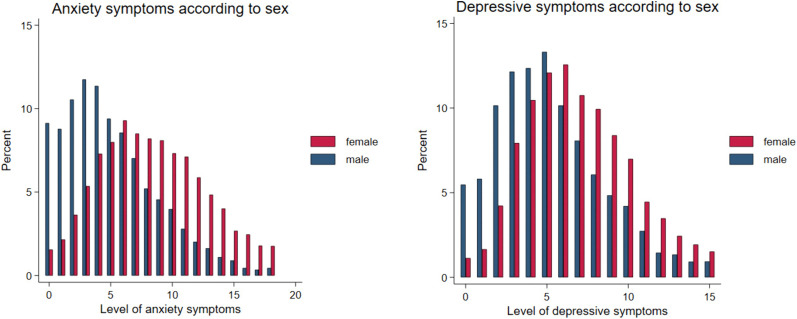
Distributions of anxiety and depressive symptom scores according to sex.

Anxiety and depressive symptom scores were higher for female participants than male participants (Table 3). To compare these differences, scores were transformed to standard ‘z’ scores. The sex difference was more pronounced for anxiety (0.75 standard deviation units) than depressive symptoms (0.49 standard deviation units). Distributions for both variables were slightly positively skew, but show a shift along the entire distributions, with female participants being consistently over-represented at the higher ends (Fig 1).

**Fig 2:**
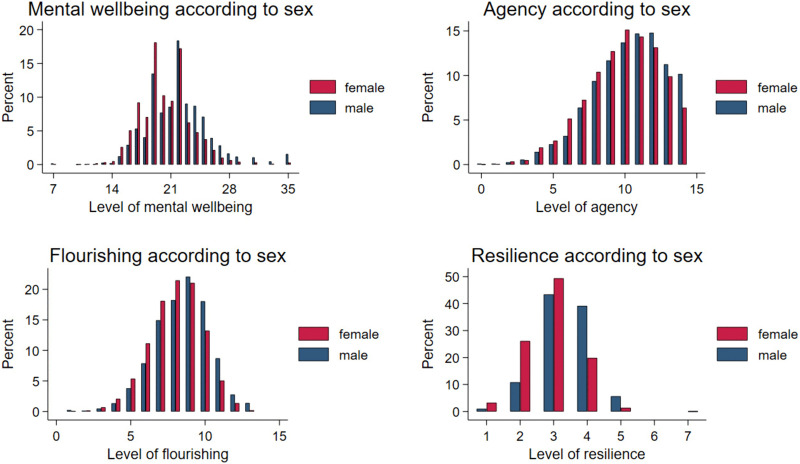
Distributions of wellbeing scores according to sex.

For wellbeing, similar but less pronounced sex differences were found, with females reporting slightly lower scores across the distribution for overall wellbeing and flourishing (Fig 2). For resilience, male participants were over-represented at the high end of the distribution. Males being more ‘agentic’ was largely due to boys being substantially over-represented at the top end of the scale.

### Lifestyle

**Fig 3:**
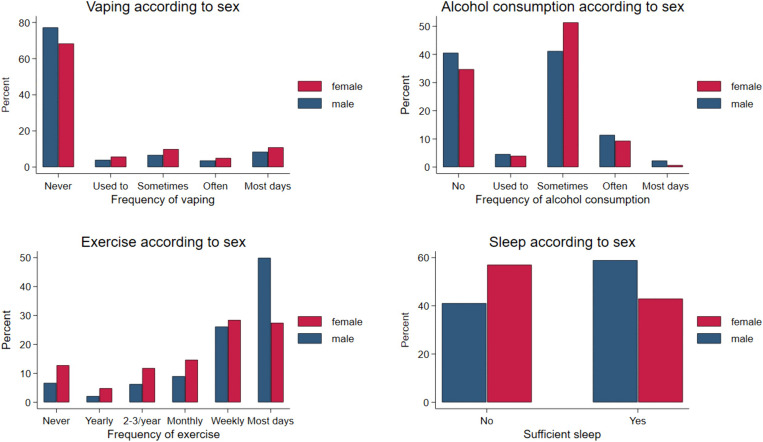
Distributions of lifestyle scores according to sex.

The prevalence of vaping was around 23% and more frequent among female (≈26%) participants (Table 3). This is in contrast to smoking where a prevalence of ≈12% was found for both sexes. Female participants were more likely to consume alcohol, whilst males were more likely to take drugs, and exercise. However, the distributions are more informative (Fig 3). For vaping, higher rates in females occur across the distribution, whereas alcohol consumption has a higher frequency for both sexes in the mid-distribution, with males being over-represented at both ends. For exercise, males are far more likely to exercise daily. Male participants were also more likely to report having sufficient sleep.

### Social media

**Fig 4:**
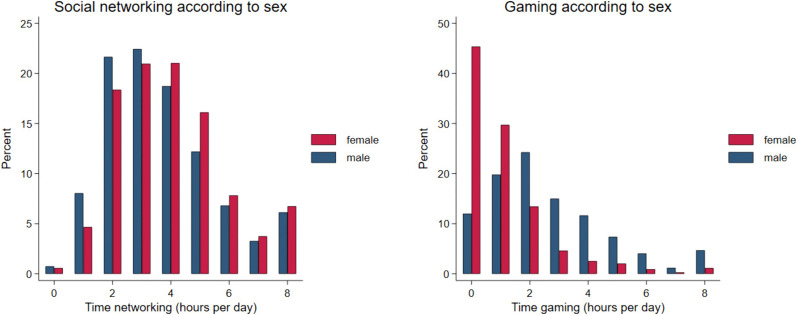
Distributions of social media use according to sex.

Social media use as hours per day was positively skew for time spent gaming and networking. Although time spent networking was greater for females (Table 3), the shape of the distributions was similar for both sexes; there being an up-tick for reporting 8 hours or more (Fig 4). This up-tick suggests there is a small group (≈7%) for whom networking has specific value, beyond that held by the wider population. Time spent gaming was substantially greater for male participants, with 17.3% spending more than 4 hours a day gaming compared with 4.3% for female participants. This distribution was positively skew with 45% of females not reporting any gaming. A small uptick was also noticed for engaging in gaming for 8 hours a day or more.

### External validity

Sleep and exercise were strong predictors of flourishing, wellbeing, anxiety and depression, with sleep being positively associated with flourishing (b = 13.71 [95% CI: 13.11, 14.31], p < .001) and wellbeing (b = 2.57 [2.46, 2.69], p < .001), and inversely associated with anxiety (b = −3.06 [−3.20, −2.91], p = < .001) and depressive symptoms (b = −3.15 [−3.25, −3.04], p < .001). Similarly, exercise was positively associated with flourishing (b = 2.85 [2.65, 3.04], p < .001) and wellbeing (b = .43 [.40,.47], p < .001), and inversely associated with anxiety (b = −.57 [−.62, −.52], p < .001) and depressive symptoms (b = −.48 [−.52, −.44], p < .001).

Alcohol consumption was inversely associated with flourishing (b = −1.23 [−1.52, −.94], p < .001) and wellbeing (b = −.21 [−.26, −.15], p < .001), and positively associated with anxiety (b = .35 [.28,.42], p < .001) and depressive symptoms (b = .33 [.28,.39], p < .001). Vaping was also associated with lower flourishing (b = −2.35 [−2.59, −2.12], p < .001) and wellbeing scores (b = −.35 [−.40, −.31], p < .001) and with higher anxiety (b = .46 [.40,.52], p < .001) and depressive symptoms (b = .50 [.46,.55], p < .001).

Smoking was inversely associated with flourishing (b = −3.32 [−3.68, −2.96], p < .001) and wellbeing (b = −.46 [−.52, −.39], p < .001), and positively associated with anxiety (b = .61 [.52,.70], p < .001) and depressive symptoms (b = .66 [.60,.73], p < .001).

Finally, higher social media use (hours per day) for social networking was inversely associated with flourishing (b = −1.89 [−2.07, −1.70], p < .001) and wellbeing (b = −.32 [−.36, −.29], p < .001), and positively associated with anxiety (b = .38 [.33,.42], p < .001]) and depressive symptoms (b = .38 [.35,.41], p < .001).

## Discussion

The feasibility of recruiting at scale and pace to an adolescent mental health and wellbeing cohort study has been demonstrated. Over the data collection period, from a population of 33,531 students aged 16 + years in 36 schools, 15,444 (46%) consented to participate, of whom 97% provided some data, 60% consented to linking their research data to educational records, and 39% to attendance records. The data indicate sex differences for anxiety, depression, sleep, and exercise. Associations of lifestyle with mental health and wellbeing were also found.

The large sex difference in mental health scores is consistent with reports in previous studies [7,14]. However, this did not translate into large sex differences in wellbeing scores, suggesting that distinct pathways are operating, perhaps comparable to those found with positive and negative emotions [15]. For the wellbeing scales, data are scarce, but the negative skew on the flourishing scores also follow similar patterns to a previous study involving younger adolescents [16]. The association of physical activity and mental health is fairly well established in both adults [17] and young people [18,19]. In our study, the bivariate inverse associations between physical activity and both depression and anxiety were modest, despite being highly significant (p < 0.001). This suggests cognitive benefits of physical activity are part of a wider picture. Our findings on sleep and wellbeing also support those found elsewhere [20,21]. Unsurprisingly, sleep is good for you. Of interest for the design of stratified trials is the reciprocity of this relationship.

### Acceptability

The rationale and logistics of working together with schools to deliver a major scientific project was successful insofar as 36 from 41 schools participated. An ‘education-led’ recruitment model was effective in attracting teachers with an interest in mental health and wellbeing through a series of professional development webinars, as well as lessons that could be used within the school’s PSHE curriculum. By working with schools to arrange a specific day (or days) for pupils to conduct the questionnaire, the time demands on schools, although significant, were reduced as much as possible, and the sense of partnership and relevance to the school mission enhanced. This sense of partnership was reinforced by providing schools with a summary profile of their pupils as a group compared with the whole cohort, following the conclusion of the study. Engagement with the BrainWaves YPAG to co-design the questionnaire also appears to have been beneficial in that the questionnaire was widely acceptable. Non-completion was monotonically related to the length of the questionnaire and appeared to be largely due to fatigue, rather than non-compliance.

### Sampling bias

The study is not designed to deliver a representative population sample, but to recruit a diverse and heterogeneous sample that is inclusive of different population strata. Heterogeneity is largely constrained by the demography of participating schools. Our attempts to recruit a diverse selection of school types across England and Scotland were reasonably successful in that the proportion of White students (76%) recruited is reasonably close to the national average for England and Wales (82%) [22]. For specific ethnic minorities, 10% for the Asian community and 3% for the African/African-Caribbean community are comparable to national figures: 9% for Asian and 4% for African/African-Caribbean [22].

The analysis of missingness according to module sequence demonstrates differential ‘drop-out’ according to sample characteristics. Although this raises practical issues of module length and sequence, no solution is cost free. Missingness can be viewed in two ways. At one level it introduces sampling bias; a factor that cannot be ignored. However, a cohort study can also be considered the direct analogue of the experiment, in the sense that it is the investigator who selects subjects to observe and who classifies these subjects according to exposures [23]. In this sense, albeit with its own execution challenges, the cohort provides a uniquely comprehensive and versatile design by which realistically complex and emerging questions can be addressed. In this context, factors determining missingness are informative. Our analysis of missingness was designed to show whether the missingness was at random, rather than provide a detailed treatment of why it was not. Nevertheless, the latter would be instructive, for discovery as well as design applications.

### Information bias

The extent to which our digital data collection method gave comparable scores to other methods, such as interview or paper questionnaire, is moot due to a paucity of data. Peer-reviewed direct methodology comparisons are rare in population studies and we are unaware of any involving adolescents. One study in older people, however, found no material difference in life satisfaction, self-esteem, self-efficacy and cognitive performance scores between clinic-based data collection and remote digital data collection [24]. The slightly positive skew of the distributions for anxiety and depressive symptom scores follow those of other studies [25,26].

### Public health messaging

The associations reported are illustrative of the challenges surrounding translating findings into policy. That sleep, exercise, and agency are associated with higher wellbeing and better mental health is consistent with previous studies [21,27–29]. These findings, however, do not necessarily imply causation. Rather, they indicate a direction of travel; an encouragement to address the complexity of adolescence in greater detail and a basis for designing and testing interventions.

### Cost-effectiveness

Precise cost modelling is challenging due to the large number of determinants and externalities involved. For example, it is more cost-effective to recruit a larger sixth form college than a smaller school. Also, many hard-to-reach groups are under-represented in large main-stream schools. In terms of planning and infrastructure, much of the informatics cost is front loaded, so there is an incremental reduction in cost per participant with increasing study size. Nevertheless, a cost of ≈£20 per recruited participant is a reasonable estimate.

### Limitations and challenges

The study has several limitations. The age of participants being limited to 16 + year olds simplified the consent process, as students were able to provide their own consent. As this pilot was primarily designed to test the school recruitment pipeline, questionnaire design and the informatics-led data collection, the age constraint was a pragmatic compromise. Nevertheless, issues surrounding parental assent and questionnaire development for younger age groups remain to be addressed. This process is underway.

Our 46% response rate cannot be considered as representative of UK 16-year-olds. Although the sampling frame was all eligible students on the schools’ rolls, in practice it was students who attended on the day of data collection. It is likely that students with poor mental health will be under-represented due to school absence. Nevertheless, achieving a 46% response rate suggests that these data offer preliminary insights into national trends in wellbeing and mental health, as well as their determinants.

Reducing the participant burden is an obvious strategy to improving response rate. For this proof-of-concept study, standard questionnaires were used. However, legacy questionnaires are frequently psychometrically inefficient. The use of classical test theory in questionnaire development offers marginal gains in validity and precision with additional items. For smaller tightly-focused studies, these gains can be considered ‘cost’ effective. For large population studies with broad health application, including relatively uninformative items is sub-optimal due to increased power from sample size and the impact on participant burden.

Although the scientific value of at-scale heterogeneous cohorts such as BrainWaves has been demonstrated elsewhere [30], this does not diminish the validity and importance of representative sampling. Larger heterogeneous and smaller representative samples serve different purposes. The former is a cost-effective means of investigating aetiology, whilst the latter is a cost-effective means of informing the public health; they are synergistic but can be unhelpfully conflated.

A major challenge is retention. High follow-up rates are critical to causal inference. For young people, we do not have the technologies or incentives to achieve high active follow-up rates by re-contact once they have left school. Lowering the age range of BrainWaves will provide increased opportunity for school-based active follow-up. However, BrainWaves does have consent for passive follow-up through access to educational records. As a general principle, augmenting research data with administrative data such as education and health records is a cost-effective means of achieving near complete follow-up. Establishing national pipelines for augmentation would be transformative for UK cohort studies.

A further challenge is safeguarding. A safeguarding flagging rate of 3% leading to a request for assistance rate of 0.4% may be interpreted variously. Although reporting rates are low, under-reporting is likely, as is under-representation of vulnerable groups. Did participation adversely affect wellbeing? Evidence on this is sparse. No objections to the questions being asked were received and the incremental pattern of module non-completion does not suggest any particular module to be associated with reduced participation. However, addressing sensitive subjects, particularly around self-harm and adversity/maltreatment require care to collect important data without suggesting harmful behaviour.

### Data access

To maximise scientific value, these data can be accessed on request via the BrainWaves Informatics Hub; a trusted research environment hosted by Dementias Platform UK. Details on making an access request can be found on the BrainWaves website (https://brainwaveshub.org/for-research/).

### Further work

BrainWaves has been designed to enable high-volume, cost-efficient, and secure data collection and access. From the cohort perspective, this includes lowering the age range and extending consent to include linkage to health records. It also includes using these data to develop more psychometrically efficient assessment tools. A major challenge is improving response rates for recruitment and retention. Whilst Toledano, Smith [31] describe several strategies for incentivising adolescent recruitment, retention remains a major challenge in this age group. A further round of data collection to increase the sample size is planned for Autumn 2025.

For schools, the programme of lessons is being extended. A study to evaluate the impact of BrainWaves lessons is planned. Work is also required to improve the value-proposition to schools in terms of data access and educational support. Whilst BrainWaves enjoys strong school support, it is important that the partnership is developed to meet the needs of schools in a demanding and changing educational environment. Of particular interest is developing an interactive tool enabling young people to interrogate BrainWaves data for themselves to encourage discovery and critical thinking at school and home.

BrainWaves has also been designed to create opportunity. Building the study around a secure informatics hub, and in partnership with schools, is a model that can be copied and improved on elsewhere. Of particular interest is using this model for a family-based study to provide inter-generational data. BrainWaves data are now available for stratified recruitment to nested sub-studies including individual and cluster randomised trials. We invite proposals for these nested studies. Our Informatics Hub is available for use by other research groups, either as a data management solution for existing data, or as a data collection and management tool for independent projects. The Informatics Hub provides a non-duplicative, ecologically supportive, cost-effective data management solution for cohorts and trials in other regions and contexts, particularly for populations in the Global South. We welcome discussions on how the study of adolescent wellbeing and mental and physical health can be promoted more widely.

## Supporting information

S1 Table Bivariate regression output (illustrative associations). (PDF)
