# Supplementary material for: The BrainWaves study of adolescent wellbeing and mental health: Methods development and pilot data
Source: PLoS One. 2025 Dec 2;20(12):e0338009. doi: 10.1371/journal.pone.0338009 (PMC12671792; doi:10.1371/journal.pone.0338009)
Supplement: S1 Table — (PDF) [file pone.0338009.s001.pdf]

```
      name:  <unnamed>
       log:  S:\0805 - The BrainWaves cohort study preliminary analysis\ryan_w
> orking_folder\POC\POC Regression.smcl
  log type:  smcl
 opened on:  26 Feb 2025, 11:12:43

1 . do "C:\Users\rparsons\AppData\Local\Temp\STD24d8_000000.tmp"

2 . regress flourish sleep

      Source |       SS           df       MS      Number of obs   =    12,046
-------------+----------------------------------   F(1, 12044)     =   1996.69
       Model |  566081.423         1  566081.423   Prob > F        =    0.0000
    Residual |  3414590.02    12,044  283.509633   R-squared       =    0.1422
-------------+----------------------------------   Adj R-squared   =    0.1421
       Total |  3980671.45    12,045  330.483308   Root MSE        =    16.838

------------------------------------------------------------------------------
    flourish | Coefficient  Std. err.      t    P>|t|     [95% conf. interval]
-------------+----------------------------------------------------------------
       sleep |   13.71164   .3068554    44.68   0.000     13.11015    14.31312
       _cons |   69.50671   .2154793   322.57   0.000     69.08434    69.92909
------------------------------------------------------------------------------

3 . regress flourish smoke

      Source |       SS           df       MS      Number of obs   =    12,019
-------------+----------------------------------   F(1, 12017)     =    321.12
       Model |  102993.338         1  102993.338   Prob > F        =    0.0000
    Residual |  3854243.53    12,017  320.732589   R-squared       =    0.0260
-------------+----------------------------------   Adj R-squared   =    0.0259
       Total |  3957236.87    12,018  329.275825   Root MSE        =    17.909

------------------------------------------------------------------------------
    flourish | Coefficient  Std. err.      t    P>|t|     [95% conf. interval]
-------------+----------------------------------------------------------------
       smoke |  -3.321064   .1853294   -17.92   0.000    -3.684339   -2.957789
       _cons |   77.40795   .1748319   442.76   0.000     77.06525    77.75064
------------------------------------------------------------------------------
```

4 . regress flourish alc

Source	SS	df	MS
Model	22550.8233	1	22550.8233
Residual	3935580.58	12,018	327.473838
Total	3958131.4	12,019	329.322856

Number of obs	=	12,020
F(1, 12018)	=	68.86
Prob > F	=	0.0000
R-squared	=	0.0057
Adj R-squared	=	0.0056
Root MSE	=	18.096

flourish	Coefficient	Std. err.	t	P>|t|	[95% conf.	interval]
alc	-1.226046	.1477453	-8.30	0.000	-1.51565	-.936441
_cons	77.9231	.2591323	300.71	0.000	77.41516	78.43104

5 . regress flourish vape

Source	SS	df	MS
Model	120824.911	1	120824.911
Residual	3829390.4	11,999	319.142462
Total	3950215.31	12,000	329.184609

Number of obs	=	12,001
F(1, 11999)	=	378.59
Prob > F	=	0.0000
R-squared	=	0.0306
Adj R-squared	=	0.0305
Root MSE	=	17.865

flourish	Coefficient	Std. err.	t	P>|t|	[95% conf.	interval]
vape	-2.353385	.1209503	-19.46	0.000	-2.590468	-2.116303
_cons	78.05308	.1869019	417.62	0.000	77.68672	78.41944

6 . regress flourish exercise

Source	SS	df	MS
Model	252519.089	1	252519.089
Residual	3647414.98	11,864	307.435517
Total	3899934.07	11,865	328.692294

Number of obs	=	11,866
F(1, 11864)	=	821.37
Prob > F	=	0.0000
R-squared	=	0.0647
Adj R-squared	=	0.0647
Root MSE	=	17.534

flourish	Coefficient	Std. err.	t	P>|t|	[95% conf.	interval]
exercise	2.845223	.0992765	28.66	0.000	2.650625	3.039821
_cons	66.23449	.3848232	172.12	0.000	65.48017	66.9888

```
7 . regress flourish time_networking

      Source |       SS           df       MS      Number of obs   =    10,320
-------------+----------------------------------   F(1, 10318)     =    394.50
       Model |  125379.957         1  125379.957   Prob > F        =    0.0000
    Residual |  3279255.79    10,318  317.818937   R-squared       =    0.0368
-------------+----------------------------------   Adj R-squared   =    0.0367
       Total |  3404635.74    10,319  329.938535   Root MSE        =    17.827

------------------------------------------------------------------------------
> ---
       flourish | Coefficient  Std. err.      t    P>|t|     [95% conf. interv
> al]
----------------+-------------------------------------------------------------
> ---
time_networking |  -1.886579   .0949841   -19.86   0.000    -2.072766   -1.700
> 392
          _cons |   83.31529   .4055998   205.41   0.000     82.52023     84.11
> 034
------------------------------------------------------------------------------
> ---

8 .
9 . regress swemwbs sleep

      Source |       SS           df       MS      Number of obs   =    12,360
-------------+----------------------------------   F(1, 12358)     =   2050.79
       Model |  20482.9231         1  20482.9231   Prob > F        =    0.0000
    Residual |  123429.677    12,358  9.98783599   R-squared       =    0.1423
-------------+----------------------------------   Adj R-squared   =    0.1423
       Total |    143912.6    12,359  11.6443564   Root MSE        =    3.1604

------------------------------------------------------------------------------
     swemwbs | Coefficient  Std. err.      t    P>|t|     [95% conf. interval]
-------------+----------------------------------------------------------------
       sleep |   2.574868   .0568584    45.29   0.000     2.463417    2.686319
       _cons |   19.66881   .0399373   492.49   0.000     19.59052    19.74709
------------------------------------------------------------------------------
```

10 . regress swemwbs smoke

Source	SS	df	MS			
				Number of obs	=	12,339
				F(1, 12337)	=	172.47
Model	1975.00781	1	1975.00781	Prob > F	=	0.0000
Residual	141270.84	12,337	11.4509881	R-squared	=	0.0138
				Adj R-squared	=	0.0137
Total	143245.848	12,338	11.6101352	Root MSE	=	3.3839

swemwbs	Coefficient	Std. err.	t	P>|t|	[95% conf.	interval]
smoke	-.4555164	.034685	-13.13	0.000	-.5235044	-.3875285
_cons	21.09713	.0325908	647.33	0.000	21.03325	21.16102

11 . regress swemwbs alc

Source	SS	df	MS			
				Number of obs	=	12,340
				F(1, 12338)	=	56.75
Model	655.776024	1	655.776024	Prob > F	=	0.0000
Residual	142560.672	12,338	11.5546014	R-squared	=	0.0046
				Adj R-squared	=	0.0045
Total	143216.448	12,339	11.6068115	Root MSE	=	3.3992

swemwbs	Coefficient	Std. err.	t	P>|t|	[95% conf.	interval]
alc	-.2060386	.0273494	-7.53	0.000	-.2596477	-.1524294
_cons	21.2191	.047843	443.52	0.000	21.12532	21.31288

12 . regress swemwbs vape

Source	SS	df	MS			
				Number of obs	=	12,314
				F(1, 12312)	=	245.91
Model	2794.61386	1	2794.61386	Prob > F	=	0.0000
Residual	139918.926	12,312	11.3644352	R-squared	=	0.0196
				Adj R-squared	=	0.0195
Total	142713.54	12,313	11.5904767	Root MSE	=	3.3711

swemwbs	Coefficient	Std. err.	t	P>|t|	[95% conf.	interval]
vape	-.3539993	.0225744	-15.68	0.000	-.3982486	-.30975
_cons	21.20773	.0347717	609.91	0.000	21.13957	21.27588

```
13 . regress swemwbs exercise

      Source |       SS           df       MS      Number of obs   =    12,160
-------------+----------------------------------   F(1, 12158)     =    542.80
       Model |  6005.80986         1  6005.80986   Prob > F        =    0.0000
    Residual |  134521.832    12,158  11.0644705   R-squared       =    0.0427
-------------+----------------------------------   Adj R-squared   =    0.0427
       Total |  140527.642    12,159     11.5575   Root MSE        =    3.3263

------------------------------------------------------------------------------
     swemwbs | Coefficient  Std. err.      t    P>|t|     [95% conf. interval]
-------------+----------------------------------------------------------------
    exercise |   .4333277   .0185993    23.30   0.000     .3968701    .4697852
       _cons |     19.411   .0721077   269.19   0.000     19.26966    19.55235
------------------------------------------------------------------------------

14 . regress swemwbs time_networking

      Source |       SS           df       MS      Number of obs   =    10,568
-------------+----------------------------------   F(1, 10566)     =    336.78
       Model |   3734.6722         1   3734.6722   Prob > F        =    0.0000
    Residual |  117171.493    10,566  11.0894845   R-squared       =    0.0309
-------------+----------------------------------   Adj R-squared   =    0.0308
       Total |  120906.165    10,567  11.4418629   Root MSE        =    3.3301

------------------------------------------------------------------------------
> ---
        swemwbs | Coefficient  Std. err.      t    P>|t|     [95% conf. interv
> al]
----------------+-------------------------------------------------------------
> ---
time_networking |  -.3214781   .0175178   -18.35   0.000    -.3558164    -.2871
> 398
          _cons |   22.13096   .0747783   295.95   0.000     21.98438     22.27
> 754
------------------------------------------------------------------------------
> ---
```

```
15 .
16 . regress anx sleep

      Source |       SS           df       MS      Number of obs   =    12,297
-------------+----------------------------------   F(1, 12295)     =   1724.52
       Model |  28707.4329         1  28707.4329   Prob > F        =    0.0000
    Residual |  204670.062    12,295  16.6466094   R-squared       =    0.1230
-------------+----------------------------------   Adj R-squared   =    0.1229
       Total |  233377.495    12,296  18.9799524   Root MSE        =      4.08

------------------------------------------------------------------------------
         anx | Coefficient  Std. err.      t    P>|t|     [95% conf. interval]
-------------+----------------------------------------------------------------
       sleep |  -3.056094   .0735923   -41.53   0.000    -3.200346   -2.911841
       _cons |   8.562029   .0516873   165.65   0.000     8.460713    8.663344
------------------------------------------------------------------------------

17 . regress anx smoke

      Source |       SS           df       MS      Number of obs   =    12,264
-------------+----------------------------------   F(1, 12262)     =    189.09
       Model |  3533.78999         1  3533.78999   Prob > F        =    0.0000
    Residual |  229163.154    12,262  18.6888888   R-squared       =    0.0152
-------------+----------------------------------   Adj R-squared   =    0.0151
       Total |  232696.944    12,263  18.9755316   Root MSE        =    4.3231

------------------------------------------------------------------------------
         anx | Coefficient  Std. err.      t    P>|t|     [95% conf. interval]
-------------+----------------------------------------------------------------
       smoke |   .6122352   .0445235    13.75   0.000      .524962    .6995083
       _cons |   6.846997   .0417642   163.94   0.000     6.765132    6.928861
------------------------------------------------------------------------------

18 . regress anx alc

      Source |       SS           df       MS      Number of obs   =    12,266
-------------+----------------------------------   F(1, 12264)     =    101.86
       Model |  1916.58761         1  1916.58761   Prob > F        =    0.0000
    Residual |  230764.142    12,264  18.8163847   R-squared       =    0.0082
-------------+----------------------------------   Adj R-squared   =    0.0082
       Total |   232680.73    12,265  18.9711154   Root MSE        =    4.3378

------------------------------------------------------------------------------
         anx | Coefficient  Std. err.      t    P>|t|     [95% conf. interval]
-------------+----------------------------------------------------------------
         alc |   .3535734   .0350335    10.09   0.000     .2849022    .4222445
       _cons |   6.578152   .0613236   107.27   0.000     6.457948    6.698356
------------------------------------------------------------------------------
```

```
19 . regress anx vape

      Source |       SS           df       MS      Number of obs   =    12,243
-------------+----------------------------------   F(1, 12241)     =    249.94
       Model |  4645.17804         1  4645.17804   Prob > F        =    0.0000
    Residual |  227499.206    12,241  18.5850181   R-squared       =    0.0200
-------------+----------------------------------   Adj R-squared   =    0.0199
       Total |  232144.384    12,242  18.9629459   Root MSE        =     4.311

------------------------------------------------------------------------------
         anx | Coefficient  Std. err.      t    P>|t|     [95% conf. interval]
-------------+----------------------------------------------------------------
        vape |   .4584311   .0289971    15.81   0.000     .4015922    .5152699
       _cons |   6.710071   .0445982   150.46   0.000     6.622651     6.79749
------------------------------------------------------------------------------

20 . regress anx exercise

      Source |       SS           df       MS      Number of obs   =    12,111
-------------+----------------------------------   F(1, 12109)     =    569.56
       Model |  10309.3923         1  10309.3923   Prob > F        =    0.0000
    Residual |  219180.372    12,109   18.100617   R-squared       =    0.0449
-------------+----------------------------------   Adj R-squared   =    0.0448
       Total |  229489.764    12,110  18.9504347   Root MSE        =    4.2545

------------------------------------------------------------------------------
         anx | Coefficient  Std. err.      t    P>|t|     [95% conf. interval]
-------------+----------------------------------------------------------------
    exercise |  -.5695436   .0238648   -23.87   0.000    -.6163224   -.5227649
       _cons |   9.077894   .0925933    98.04   0.000     8.896396    9.259392
------------------------------------------------------------------------------

21 . regress anx time_networking

      Source |       SS           df       MS      Number of obs   =    10,525
-------------+----------------------------------   F(1, 10523)     =    275.24
       Model |  5152.80627         1  5152.80627   Prob > F        =    0.0000
    Residual |  197004.472    10,523  18.7213221   R-squared       =    0.0255
-------------+----------------------------------   Adj R-squared   =    0.0254
       Total |  202157.278    10,524  19.2091675   Root MSE        =    4.3268
```

```
───────────────┬────────────────────────────────────────────────────────────────
> ───
           anx │ Coefficient  Std. err.      t    P>|t|     [95% conf. interv
> al]
───────────────┼────────────────────────────────────────────────────────────────
> ───
time_networking │   .3778892   .0227778    16.59   0.000     .3332405    .4225
> 379
          _cons │   5.705408   .0973144    58.63   0.000     5.514653    5.896
> 162
───────────────┴────────────────────────────────────────────────────────────────
> ───

22 .
23 . regress dep sleep

      Source │       SS           df       MS      Number of obs   =    12,332
─────────────┼──────────────────────────────────   F(1, 12330)     =   3467.81
       Model │  30563.9626         1  30563.9626   Prob > F        =    0.0000
    Residual │  108671.945    12,330  8.81362083   R-squared       =    0.2195
─────────────┼──────────────────────────────────   Adj R-squared   =    0.2194
       Total │  139235.907    12,331  11.2915341   Root MSE        =    2.9688

─────────────┬────────────────────────────────────────────────────────────────
         dep │ Coefficient  Std. err.      t    P>|t|     [95% conf. interval]
─────────────┼────────────────────────────────────────────────────────────────
       sleep │  -3.148889   .0534724   -58.89   0.000    -3.253704   -3.044075
       _cons │   7.772924   .0375554   206.97   0.000     7.699309    7.846538
─────────────┴────────────────────────────────────────────────────────────────

24 . regress dep smoke

      Source │       SS           df       MS      Number of obs   =    12,299
─────────────┼──────────────────────────────────   F(1, 12297)     =    381.35
       Model │  4156.88548         1  4156.88548   Prob > F        =    0.0000
    Residual │  134041.856    12,297  10.9003705   R-squared       =    0.0301
─────────────┼──────────────────────────────────   Adj R-squared   =    0.0300
       Total │  138198.741    12,298  11.2374972   Root MSE        =    3.3016

─────────────┬────────────────────────────────────────────────────────────────
         dep │ Coefficient  Std. err.      t    P>|t|     [95% conf. interval]
─────────────┼────────────────────────────────────────────────────────────────
       smoke │   .6625276   .0339266    19.53   0.000     .5960261    .7290291
       _cons │   5.994117   .0318493   188.20   0.000     5.931688    6.056547
─────────────┴────────────────────────────────────────────────────────────────
```

25 . regress dep alc

Source	SS	df	MS
Model	1706.66424	1	1706.66424
Residual	136717.512	12,298	11.1170526
Total	138424.177	12,299	11.2549131

Number of obs	=	12,300
F(1, 12298)	=	153.52
Prob > F	=	0.0000
R-squared	=	0.0123
Adj R-squared	=	0.0122
Root MSE	=	3.3342

dep	Coefficient	Std. err.	t	P>|t|	[95% conf.	interval]
alc	.3332884	.0268993	12.39	0.000	.2805615	.3860152
_cons	5.772879	.0470546	122.68	0.000	5.680645	5.865113

26 . regress dep vape

Source	SS	df	MS
Model	5625.38931	1	5625.38931
Residual	132406.411	12,275	10.786673
Total	138031.8	12,276	11.2440372

Number of obs	=	12,277
F(1, 12275)	=	521.51
Prob > F	=	0.0000
R-squared	=	0.0408
Adj R-squared	=	0.0407
Root MSE	=	3.2843

dep	Coefficient	Std. err.	t	P>|t|	[95% conf.	interval]
vape	.5024042	.0219999	22.84	0.000	.4592809	.5455274
_cons	5.842533	.0339542	172.07	0.000	5.775978	5.909089

27 . regress dep exercise

Source	SS	df	MS
Model	7323.00882	1	7323.00882
Residual	129414.38	12,139	10.6610413
Total	136737.389	12,140	11.2633764

Number of obs	=	12,141
F(1, 12139)	=	686.89
Prob > F	=	0.0000
R-squared	=	0.0536
Adj R-squared	=	0.0535
Root MSE	=	3.2651

dep	Coefficient	Std. err.	t	P>|t|	[95% conf.	interval]
exercise	-.479678	.0183023	-26.21	0.000	-.5155533	-.4438026
_cons	7.922137	.0709694	111.63	0.000	7.783026	8.061248

```
28 . regress dep time_networking

      Source |       SS           df       MS      Number of obs   =    10,561
-------------+----------------------------------   F(1, 10559)     =    484.96
       Model |  5241.36824         1  5241.36824   Prob > F        =    0.0000
    Residual |  114118.835    10,559  10.8077313   R-squared       =    0.0439
-------------+----------------------------------   Adj R-squared   =    0.0438
       Total |  119360.203    10,560  11.3030496   Root MSE        =    3.2875

------------------------------------------------------------------------------
> ---
            dep | Coefficient  Std. err.      t    P>|t|     [95% conf. interv
> al]
----------------+-------------------------------------------------------------
> ---
time_networking |   .3802067   .0172649    22.02   0.000     .3463642    .4140
> 492
          _cons |    4.82473   .0737513    65.42   0.000     4.680164    4.969
> 297
------------------------------------------------------------------------------
> ---

29 .
   end of do-file

30 . exit, clear
```
